# ﻿A new species of the genus *Alainites* Waltz & McCafferty, 1994 (Ephemeroptera, Baetidae) from the north of Morocco

**DOI:** 10.3897/zookeys.1176.107829

**Published:** 2023-08-29

**Authors:** Majida El Alami, Laurent Vuataz, Sara El Yaagoubi, Jean-Luc Gattolliat

**Affiliations:** 1 Université Abdelmalek Essaâdi, Faculté des Sciences, Département de Biologie, Laboratoire Ecologie, Systématique et Conservation de la Biodiversité (LESCB), Unité de Recherche Labellisée CNRST N°18. B.P.2121. Tétouan 93002, Morocco Université Abdelmalek Essaâdi Tétouan Morocco; 2 Muséum Cantonal des Sciences Naturelles, Département de Zoologie, Palais de Rumine, Place Riponne 6, CH-1005, Lausanne, Switzerland Muséum Cantonal des Sciences Naturelles, Département de Zoologie Lausanne Switzerland; 3 University of Lausanne (UNIL), Department of Ecology and Evolution, CH-1015 Lausanne, Switzerland University of Lausanne (UNIL) Lausanne Switzerland

**Keywords:** COI, endemics, Maghreb, mayflies, Rif, systematics, West Palaearctic

## Abstract

A new species of *Alainites* is described from northern of Morocco *Alainitesalbai***sp. nov.** It can be separated from the other west Palearctic species by the gill number, the spination of the distal margin of tergites, the leg setation, and the paraproct shape and spination. This species is widespread in the study area but never abundant. It prefers small to medium streams with slow flow, and does not seem to be very sensitive to pollution and water logging activities.

## ﻿Introduction

The mayfly genus *Alainites* Waltz & McCafferty, 1994 was established to encompass species previously included in the *muticus* species group of the genus *Baetis* Leach, 1815 (Waltz et al. 1994). Currently, this genus encompasses 22 species (Barber-James et al. 2013; Kaltenbach and Gattolliat 2021; Phlai-ngam et al. 2022; Yanai et al. 2022). Also, the distribution of *Alainites* is nearly limited at the Palaearctic and Oriental regions; so far, the highest diversity is found in the West Palaearctic region with eight species. Except *Alainitesmuticus* (Linnaeus, 1758) which has a wide distribution in Europe (Bauernfeind and Soldán 2012) and has also been recently reported from western Asia (Armenia, Hrivniak et al. 2018) and Iran (Bojková et al. 2018), this genus has a high endemism rate in the Mediterranean basin. During the last decades, seven species with mostly restricted distributions have been described. These are *A.oukaimeden* (Thomas & Sartori, 1992) from the Moroccan High Atlas (Thomas and Gagneur 1994; Abdaoui et al. 2010; Zuedzang Abessolo et al. 2021), *A.sadati* Thomas, 1994 distributed from West Algeria to North Tunisia (Thomas and Gagneur 1994; Zrelli et al. 2012; Benhadji et al. 2020), *A.navasi* (Müller-Liebenau, 1974) located in the Iberian Peninsula (Müller-Liebenau 1974; Alba-Tercedor 1981, 1982; Puig 1983), *A.kars* (Thomas & Kazanci, 1989) found in Turkey (Kazanci and Thomas 1989; Novikova and Kluge 1994; Kazanci 2001; Kluge and Novikova 2014) and Armenia (Hrivniak et al. 2018; Sroka et al. 2021), *A.albinatii* (Sartori & Thomas 1989) mentioned from Corsica (Sartori and Thomas 1989; Gattolliat et al. 2015; Tenchini et al. 2018), *A.bengunn* Yanai & Gattolliat, 2022 recorded from the sister island of Sardinia (Yanai et al. 2022), and *A.gasithi* Yanai & Gattolliat, 2022 found recently in Israel (Yanai et al. 2022).

The distinctive taxonomical characters of this genus at the nymphal stage were mentioned by Zrelli et al. (2012) and Sroka et al. (2021) and include a laterally compressed body, closely positioned antennae, an apical prolongation on the paraproct, and a reduced mandibular right prostheca (Müller-Liebenau 1969, 1974; Waltz et al. 1994; Fujitani et al. 2003). West Palearctic species are characterized by the presence of six pairs of gills, except for *A.muticus*, which has seven. Some larvae of *Alainites* discovered in the material collected from northern Morocco were different from the Moroccan endemic *A.oukaimeden* and from *A.muticus*. It represents the second West Palearctic species with seven pairs of gills. The specimens of this species were previously considered to belong to *A.muticus* (Dakki 1987; El Bazi et al. 2017; Khadri et al. 2017; Mabrouki et al. 2017; El Alami et al. 2022b). However, detailed morphological and genetic approaches proved that these specimens of *Alainites* noticeably differed from both the European and Asiatic lineages (Sroka 2012).

In the present study, we describe a new species of *Alainites* based on nymphs from the Rifean Mountains of Morocco. In addition, distinctive characters of western Palearctic *Alainites* species are provided. The holotype and part of the paratypes of the new species are housed in the Muséum cantonal des sciences naturelles, Lausanne, Switzerland (**MZL**); other paratypes are deposited in Laboratory of Ecology, Systematics, Conservation of Biodiversity Tetouan, Morocco (**LESCB**).

## ﻿Materials and methods

**Figure 1.**
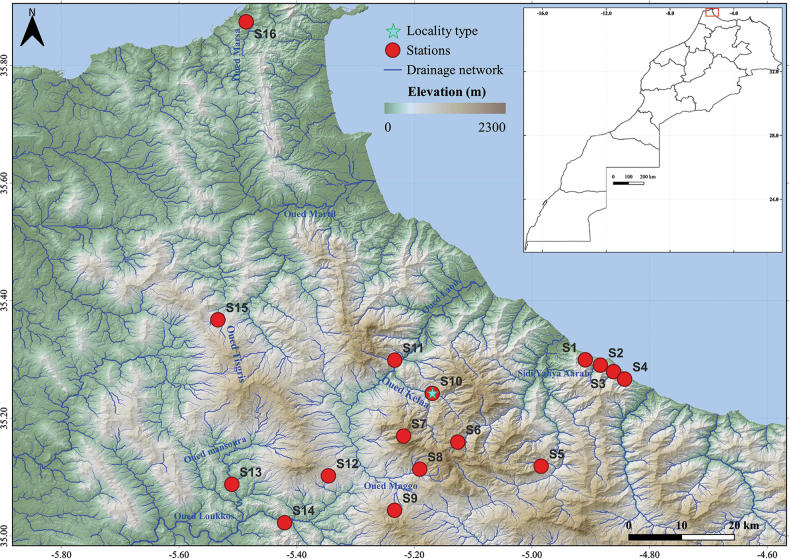
The sampling site localization of *Alainitesalbai* sp. nov., in the Rif domain.

The larvae of *Alainitesalbai* sp. nov. has a wide distribution and a wide altitudinal range in the Rif (Fig. 1). The sampling was performed by LESCB team between 1997 and 2022. They were subsequently preserved in 70% or 95% ethanol for description and DNA extraction. Nymphal dissection was performed in Cellosolve or in 10% KOH, and specimens were mounted on slides with Euparal medium, or the dissected parts of the nymphs were mounted directly in Hoyer’s liquid (Alba-Tercedor 1988), using an Olympus SZM100 stereomicroscope.

To complement our morphological investigations, we sequenced a 658 bp fragment of the mitochondrial gene cytochrome oxidase subunit 1 (COI hereafter) for seven specimens of *Alainitesalbai* sp. nov., following the non-destructive DNA extraction procedure described in Vuataz et al. 2011. The Polymerase Chain Reaction (PCR), purification and sequencing steps were conducted according to the methodology described in El Alami et al 2022a. Forward and reverse sequencing reads were assembled and edited in CodonCode Aligner v. 10.0.2 (Codon-Code Corporation, Dedham, MA), and aligned using MAFFT (Katoh et al. 2019) with default settings as implemented in Jalview v. 2.11.2.6 (Waterhouse et al. 2009). The number of parsimony-informative sites of the alignment was calculated in Mega v. 10.2.4 (Kumar et al. 2018; Stecher et al. 2020).

To augment our molecular dataset, we initially downloaded all COI sequences associated with *Alainites* available on the GenBank database (identified among species of the genus *Takobia* Novikova & Kluge, 1987) as of 2 June 2023, resulting in a total of 64 records. Additionally, we obtained the sequences associated with *Alainites*/*Takobia* accessible on the BOLDSYSTEMS data portal as of the same date and selectively retained only those that were not shared with GenBank, yielding an additional set of 5 sequences. We then manually excluded GenBank/BOLD sequences obtained from specimens collected outside the western Palearctic region. This selection process was conducted after confirming that the COI sequences of *Alainitesalbai* sp. nov. were clearly distinct from the removed sequences (data not shown). A total of 58 sequences remained for further analyses, comprising seven newly generated sequences (Table 1), 46 sequences from GenBank (four from Kjærstad et al. 2012; six from Sroka 2012; two from Cardoni et al. 2015; ten from Gattolliat et al. 2015; one from Corse et al. 2017; seven from Morinière et al. 2017; five from Tenchini et al. 2018; one from Behrens-Chapuis et al. 2021; two from Sroka et al. 2021; two from Roslin et al. 2022; five from Yanai et al 2022; one unpublished iBOL data release) and five from BOLD (DTNHM1449-21, TDAAT323-19, VMCOL019-20–VMCOL021-20). Two additional newly sequenced specimens, namely one *Nigrobaetisnumidicus* (Soldán & Thomas, 1983) from Morocco and one *Nigrobaetisrhithralis* (Soldán & Thomas, 1983) from Algeria, were included in the study as outgroups (Table 1).

**Table 1.** Newly sequenced specimens for the study, with collection information, GenBank accessions and nomenclature details.

Specimen catalogue	Species	Country	Stage	Locality	GPS coordinates	Date	GenBank ID	GenSeq nomenclature
GBIFCH00980877	*Alainitesalbai* sp. nov.	Morocco	Nymph	Oued Kelâa	35.242222°N, 5.169444°W	3.iii.2021	OR126003	genseq-2 COI
GBIFCH01144254	*Alainitesalbai* sp. nov.	Morocco	Nymph	Oued Tisgris	35.367222°N, -5.533889°W	20.iv.2021	OR126002	genseq-2 COI
GBIFCH01144390	*Alainitesalbai* sp. nov.	Morocco	Nymph	Oued Ouara	35.035039°N, -5.237778°W	23.vi.2022	OR126001	genseq-2 COI
GBIFCH01144391	*Alainitesalbai* sp. nov.	Morocco	Nymph	Oued Afeska	35.169733°N, -5.185083°W	2. vi.2020	OR126000	genseq-2 COI
GBIFCH01144392	*Alainitesalbai* sp. nov.	Morocco	Nymph	Oued Amazithen	35.29924°N, -4.90937°W	27.ii.2021	OR125999	genseq-2 COI
GBIFCH01144393	*Alainitesalbai* sp. nov.	Morocco	Nymph	Oued Ouara	35.043567°N, -5.2336°W	23.vi.2022	OR125998	genseq-2 COI
GBIFCH01144394	*Alainitesalbai* sp. nov.	Morocco	Nymph	Oued Sidi Yahia Aarab	35.287056°N, -4.90185°W	28.v.2022	OR125997	genseq-2 COI
GBIFCH00673223	*Nigrobaetisrhithralis*	Algeria	Nymph	Oued Ftitich	36.900278°N, 8.618056°W	9.iii.2019	OR126004	genseq-4 COI
GBIFCH01144576	*Nigrobaetisnumidicus*	Morocco	Nymph	Oued Brahim Ben Arrif	35.306111°N, -5.615833°W	21.vi.2013	OR125996	genseq-4 COI

To explore and visualize the COI evolutionary divergence, we employed pairwise genetic distances and gene tree approaches. COI pairwise distances were calculated using the dist.dna function from the ape 5.7-1 package (Paradis and Schliep 2019) in R 4.3.0 (R Core Team 2023), selecting the raw model and the pairwise.deletion option, corresponding to uncorrected p-distances (see Srivathsan and Meier 2012) with missing data removed in a pairwise way. Mean, minimum, and maximum distances within and between putative COI species, referred to as Molecular Operational Taxonomic Units (MOTUs) hereafter, were calculated using the ddply function from the plyr 1.8.8 package (Wickham 2011). The assignment of COI sequences to MOTUs was determined based on the results of the species delimitation analyses (as described below). Prior to reconstructing the COI gene tree, the best evolutionary model (HKY+Γ+I) was selected based on the second-order Akaike information criterion (AICc; Hurvich and Tsai 1989) implemented in JmodelTest v. 2.1.10 (Darriba et al. 2012) with three substitution schemes, six gamma categories and default values for other parameters. To account for different substitution rates among COI codon positions, we analyzed our data set in two partitions, one with first and second codon positions, and the other with third positions (1 + 2, 3). Bayesian inference analysis was performed using BEAST v. 1.10.4 (Suchard et al. 2018) on the CIPRES Science Gateway v. 3.3 (Miller et al. 2010). The input BEAST file was generated in BEAUTi v. 1.10.4 (Suchard et al. 2018), incorporating the selected evolutionary model and partition scheme described above and enforcing a monophyletic constraint on the ingroup (the genus *Alainites*). A relaxed molecular clock model (uncorrelated lognormal) and a UPGMA starting tree were used, with default settings for other parameters. Two independent Markov chain Monte Carlo (MCMC) analyses were run for 60 million generations, logging parameters every 1000 generations. Convergence of the MCMC runs was visually verified in Tracer v. 1.7.2 (Rambaut et al. 2018). The log and tree files from the independent runs were combined using LogCombiner v. 1.10.4 (Suchard et al. 2018), after discarding the initial 10% of trees as burn-in, ensuring that all parameters reached effective sample size values > 200. The maximum clade credibility tree was obtained using TreeAnnotator v. 1.10.4 (Suchard et al. 2018) with default settings. Visualization and editing of the tree were conducted in iTOL v. 6.7.5 (Letunic and Bork 2021).

Finally, we applied three contrasting single-locus species delimitation methods to our COI dataset: the distance-based ASAP (Assemble Species by Automatic Partitioning; Puillandre et al. 2020), and the tree-based GMYC (General Mixed Yule-Coalescent; Pons et al. 2006; Fujisawa and Barraclough 2013) and mPTP (multi-rate Poisson Tree Processes; Kapli et al. 2017) approaches. ASAP, an improved version of the ABGD (Automatic Barcode Gap Discovery; Puillandre et al. (2012) approach, was employed using the ASAP webserver (https://bioinfo.mnhn.fr/abi/public/asap/asapweb.html) to estimate the most probable number of MOTUs based on our COI alignment. We calculated genetic distances using the Kimura 2-parameter substitution model (Kimura 1980) and selected the species delimitation hypothesis associated to the highest barcode gap width (W) among the two partitions sharing the same best asap-score. The GMYC model, which requires a time-calibrated ultrametric tree as input, implements a maximum likelihood approach that defines a threshold separating the branches modelled under speciation events (Yule process) from those described by allele neutral coalescence. The ultrametric tree used as input for GMYC analysis was generated in BEAST, following the same procedure described earlier. However, a reduced dataset was utilized, in which outgroups were excluded and haplotypes were pruned (see Talavera et al. 2013) using Collapsetypes v. 4.6 (Chesters 2013). MCMC chains were run here for a total of 20 million generations. GMYC was run in R using the SPLITS package 1.0-20 (Ezard et al. 2009). We favored the single-threshold version of the GMYC model because it was shown to outperform the multiple-threshold version (Fujisawa and Barraclough 2013). The mPTP approach, an extension of the PTP method by Zhang et al. (2013), also exploits phylogenetic differences within and between species, but with the advantage of directly using the number of substitutions from a phylogenetic tree, eliminating the need for time calibration. We conducted mPTP using the web service available at https://mptp.h-its.org, using the BEAST COI gene trees (full dataset) as input (see above).

### ﻿Abbreviations

**MZL**Muséum Cantonal des Sciences Naturelles, Lausanne (Switzerland);

**LESCB** Laboratoire d’Ecologie, Systématique et Conservation de la Biodiversité (Morocco).

## ﻿Results

### ﻿ Alainites albai

Taxon classificationAnimaliaEphemeropteraBaetidae

El Alami, Vuataz & Gattolliat sp. nov.

1266EF47-5E89-5C65-8629-080D4804BD3D

https://zoobank.org/0B24CF09-A193-42A2-9A9C-96266B5C0C4A

Figs 1 , 2 , 3 , 4 , 5 , 6 , 7 , 8

#### Type-material.

***Holotype*.** Morocco. Nymph; Chefchaouen Province, S10 Oued Kelâa; Loc. Akchour; 35°14'32"N, 05°10'10"W; alt. 460 m; 3.iii.2021; El Yaagoubi leg.; GBIFCH00763782; MZL.

***Paratypes*.** Morocco. 1 nymph; same data as holotype; DNA; GBIFCH00980877; MZL • 2 nymphs; same data as holotype; 7.xi.2014; El Bazi leg.; in alcohol; LESCB • 1 nymph; Chefchaouen Province, S1 Oued Amazithen, Loc. El Ouesteyine; 35°17'57.264"N, 4°54'33.732"W; alt. 166 m; 27.ii.2021; El Yaagoubi leg.; DNA; GBIFCH01144392; MZL • 2 nymphs; Chefchaouen Province, S2 Oued Sidi Yahia Aarab, Loc. Sidi Yahia Aarab; 35°17'10.428"N, 4°53'37.644"W; alt. 114 m; 28.v.2022; El Yaagoubi leg.; one in alcohol; GBIFCH00763784 and other DNA; GBIFCH01144394; MZL; 1 nymph; 18.vi.2014; Khadri leg.; in Alcohol; GBIFCH00763781; MZL; 4 nymphs; 28/v/2022; El Yaagoubi leg.; LESCB • 6 nymphs ; Chefchaouen Province, S3 Oued Jenane Niche, Loc. Jenane Niche ; 35°16'44.904"N, 4°51'40.788"W ; alt. 93 m; 9.ix.2021 ; El Yaagoubi leg.; LESCB • 11 nymphs; Chefchaouen, Province, S4 Oued Aârkob, Loc. Arherarose; 35°15'59.4"N, 4°50'33.216"W; alt. 128 m; 9.xii.2021; El Yaagoubi leg.; in alcohol; LESCB • 22 nymphs; Chefchaouen Province, S5 Oued Assifane, Loc. Igourain; 35°7'6.7584"N, 4°59'3.9984"W; alt. 1405 m; 9.ix.2021; El Yaagoubi leg ; 1 on slide ; LESCB • 5 nymphs; Chefchaouen Province, S6 Oued Beni Mhammed, Loc. Beni Mhammed; 35°09'34.0812"N, 5°07'34.0212"W; alt. 1330 m; 29.v.2008; El Alami leg.; in alcohol; GBIFCH00763777; MZL • 2 nymphs; Chefchaouen Province, S7 Oued Afeska, Loc. Afeska; 35°10'11.0388"N, 5°13'6.2988"W; alt. 1293 m; 2.vi.2021; El Yaagoubi leg.; 1 nymph DNA; GBIFCH01144391; 2.vi.2020;2 in alcohol; GBIFCH00763777; GBIFCH00763783; MZL and 4 nymphs; 2.vi.2022; El Yaagoubi leg.; 3 in alcohol and 1 on slide; LESCB • 10 nymphs; Chefchaouen Province, S8 Oued Maggo, Loc. Maggo Nord village; 35°6'48.6"N, 5°11'26.7"W; alt. 905 m; 24.ii.2022; El Yaagoubi leg.; LESCB; 5 nymphs; 3.vi.2016; El Alami, leg.; in alcohol; GBIFCH00763780; MZL • 4 nymphs; Chefchaouen Province, S9 Oued Ouara, Loc. Khizana; 35°02'614"N, 5°14'016"W; alt. 930 m; 23.vi.2022; El Yaagoubi leg.; 2 on slide; LESCB; 2 for DNA; GBIFCH01144393; GBIFCH01144390; MZL • 3 nymphs; Chefchaouen Province, S11 Oued Laou, Loc. Abiyati; 35°17'55.14"N, 5°13'59.99"W; alt. 140 m; 11.iii.2001; El Alami leg.; in alcohol; GBIFCH00763779; MZL • 1 nymph; Chefchaouen Province, S12 Oued Harakat, Loc. Mezine village; 35°6'8"N, 5°20'46"W; alt. 740 m; 31.iii.2021; El Yaagoubi leg.; DNA, GBIFCH00980924; MZL • 20 nymphs; Chefchaouen Province, S13 Oued Mansoura, Loc. Tanaqoub; 35°5'16"N, 5°30'37"W; alt. 124 m; 01.vi.2021; El Yaagoubi leg.; 2 on slide; LESCB • 2 nymphs; Ouezzane Province, S14 Oued Loukkos, Loc. Souk El Had; 35°01'21"N, 5°25'14"W; alt. 140 m; 11.iv.2021; El Yaagoubi leg.; in alcohol; GBIFCH00763776; MZL • 1 nymph; Tetouan Province, S15 Oued Tisgris, Loc. Hammadesh; 35°22'02"N, 5°32'02"W; alt. 505 m; 20.iv.2021; El Yaagoubi leg.; DNA; GBIFCH01144254; MZL • 10 nymphs; Tetouan Province, S16 Oued Rmel, Loc. Ain Dchicha; 35°52'40"N, 5°28'24"W; alt. 49 m; 20.x.1997; El Alami leg.; in alcohol; LESCB and 2 nymphs in alcohol; GBIFCH00763778; MZL.

#### Differential diagnosis.

*Alainitesalbai* sp. nov. can be distinguished from other West Palaearctic species of *Alainites* based on the combination of nymphal characters, summarized in Table 2: (a) seven pairs of abdominal gills, (b) paraproct prolongation covered with small spines on its entire surface with broad, triangular spines laterally, (c) small teeth between prostheca and mola on both mandibles, (d) low number of dorsal setae on its fore-femora (10–15).

**Table 2.** Distinctive taxonomic criteria and distribution of West Palaearctic *Alainites* species.

Species	Distribution	Number of gill pairs	Right mandible: margin between prostheca and mola	Mandible lateral side	Fore-femur dorsal margin: setae number	Fore-tibia dorsal margin: setae number	Cuticle abdominal terga and sterna	Tergite IV: spines on distal margin	Prolongation of paraproct
*Alainitesalbai* sp. nov.	Morocco	7	10–16 small teeth	shagreened with scattered fine setae and deep scale bases	10–15	6–10	strongly shagreened	long triangular, pointed	covered by spines
*Alainitesalbinatii* (Sartori & Thomas, 1989)	Corsica	6	~ 10 small teeth	scale bases shagreened	15	6	slightly shagreened	long triangular, pointed	apically covered by spines
*Alainitesbengunn* Yanai & Gattolliat, 2022	Sardinia	6	serrated	scale bases, slightly shagreened	14–20	9–17	shagreened	slightly lanceolate	covered by spines
*Alainitesgasithi* Yanai & Gattolliat, 2022	Israel	6	serrated	no scale bases, almost not shagreened	10–20	~ 6 rarely 10–12	smooth	long triangular, pointed	spines only on border
*Alainiteskars* (Thomas & Kazanci, 1989)	Turkey	6	teeth absent	no scale bases, almost not shagreened	> 40 in two rows	5–9	slightly shagreened	triangular pointed	spines on entire surface or just on apex
*Alainitesmuticus* (Linnaeus, 1758)	Palaearctic	7	~ 10 small teeth	rare scale bases	14	8	slightly shagreened	short triangular broad basally	spines only on border
*Alainitesnavasi* (Müller-Liebenau, 1974)	Iberian Peninsula	6	~ 10 small teeth	?	26	21	smooth	short triangular	covered by spines
*Alainitesoukaimeden* (Thomas & Sartori, 1992)	Morocco (High Atlas)	6	~ 10 small teeth	shagreened	20	8	strongly shagreened	long, relatively narrow	covered by spines
*Alainitessadati* Thomas, 1994	Algeria, Tunisia	6	~ 10 small teeth	no scale bases, almost not shagreened	~ 25	6–9	slightly shagreened	medium triangular	covered by spines

#### Morphological description.

***Nymph*.** Length. Female body 6.0–7.9 mm; cerci 4.5–5.5 mm; median caudal filament 1.3–1.4 mm (ca 2/3 of cerci); Male body 6.0–6.7 mm; cerci 4.0–5.0 mm; median caudal filament ca 2/3 of cerci.

#### Coloration.

**Figure 2.**
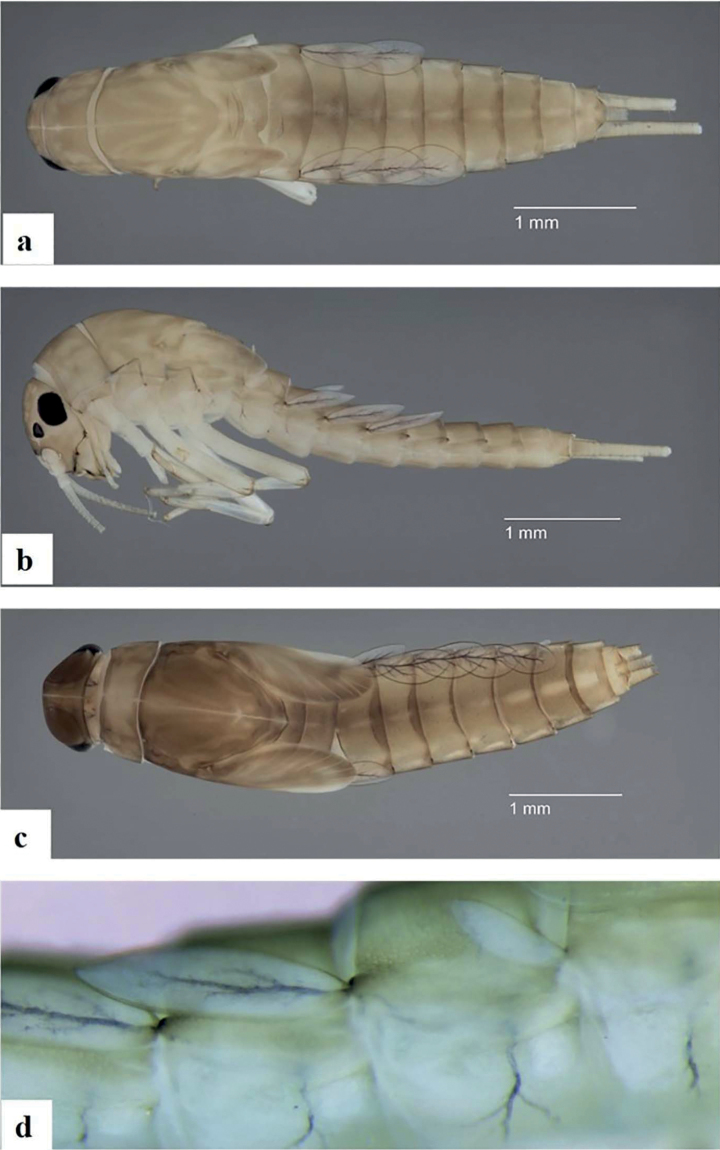
*Alainitesalbai* sp. nov., nymph habitus **a** female, dorsal view **b** female lateral view **c** male, dorsal view **d** lateral view of the first three gills.

**Figure 3.**
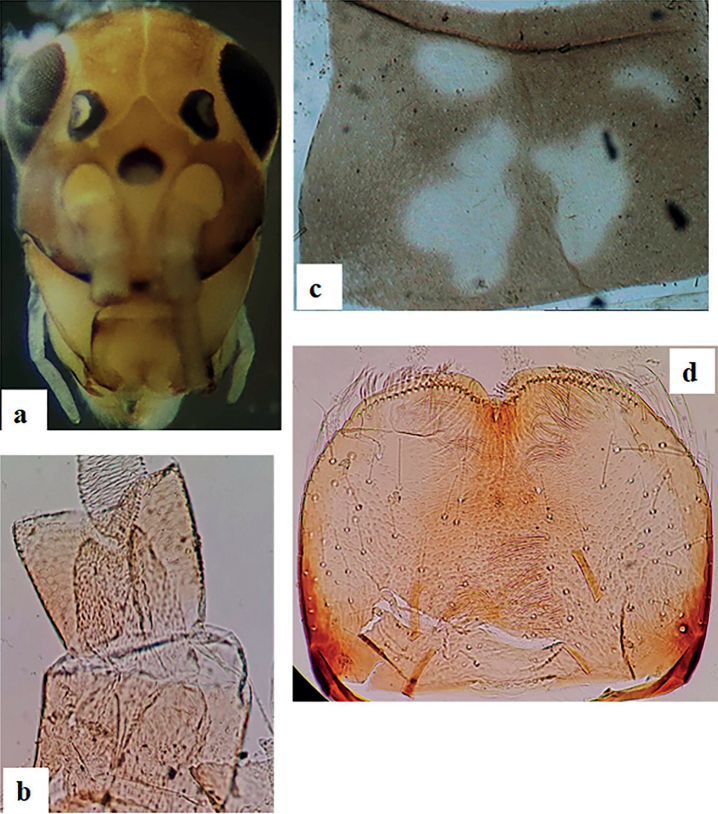
*Alainitesalbai* sp. nov., nymph morphology **a** head frontal view **b** scape and pedicel **c** pronotum **d** labrum.

General coloration pale to medium brown (Figs 2, 3). Head uniformly pale brown to brown with yellow vermiform marks on vertex and frons (Fig. 3a). Turbinate eyes in male nymph’s purple-brown. Legs ecru except upper side of femora brown (Fig. 2b). Thorax with some paler clear pattern (Fig. 3c). Abdominal tergites pale brown with a central, elongated, yellowish dot; distal part of tergite IX and whole tergite X pale brown to yellowish. Abdominal sternites yellowish to pale brown. Cerci ecru to pale brown without bands or pattern.

**Head. *Antennae*** (Fig. 3a) close to each other, with a narrow inter-antennal carina, scape with deep scale insertions, pedicel with deep scale insertions and few setae (Fig. 3b).

Dorsal surface of labrum (Fig. 3d) with one central long seta and distolateral arc of two long, simple, stout setae, and small fine setae scattered on surface; ventral surface with 5–8 submarginal small, pointed setae; distal margin fringed with ca 20 short, followed by eight or ten long, feathered setae.

**Figure 4.**
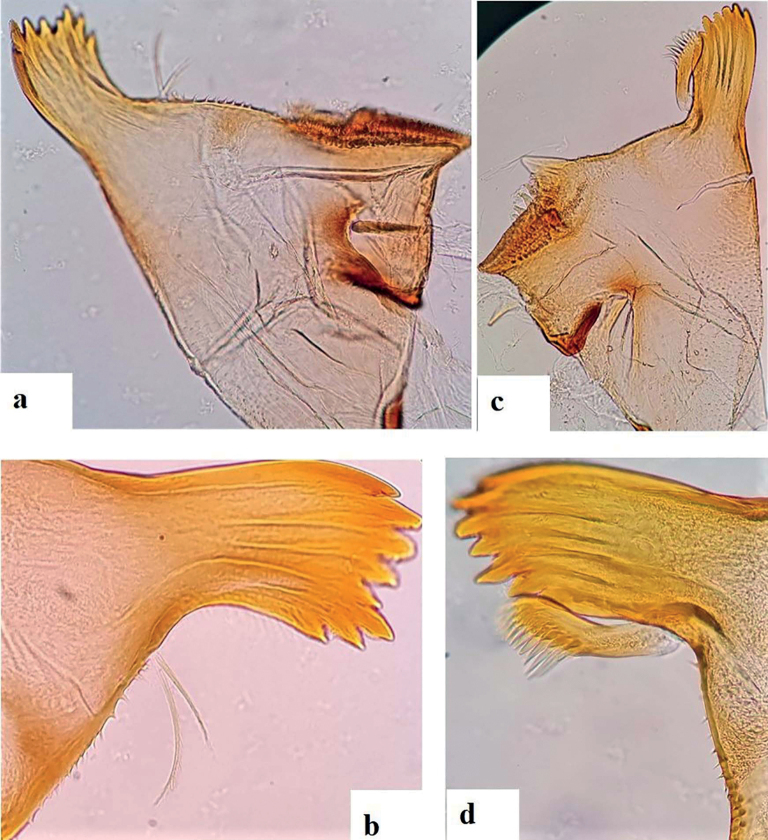
*Alainitesalbai* sp. nov., nymph morphology **a** right mandible **b** spines between mola and prostheca bifid **c** left mandible **d** spines between mola and prostheca.

***Right mandible*** (Fig. 4a) shagreened, with scattered fine setae and deep scale insertions; incisors composed of eight blunt, distinct denticles, outer- and inner denticles notably smaller than others; prostheca reduced and bifid with numerous thin setae; 10–16 teeth in the space between prostheca and mola (Fig. 4b), tuft of setae absent.

***Left mandible*** (Fig. 4c) slightly shagreened, with sparse fine setae and deep scale insertions; incisors composed of seven blunt, distinct denticles, outer denticle conspicuously smaller than others; prostheca with one row of 11–15 medium denticles and a comb-like structure (Fig. 4d); edge between prostheca and mola with sparse spines particularly in apical half, without setae.

**Figure 5.**
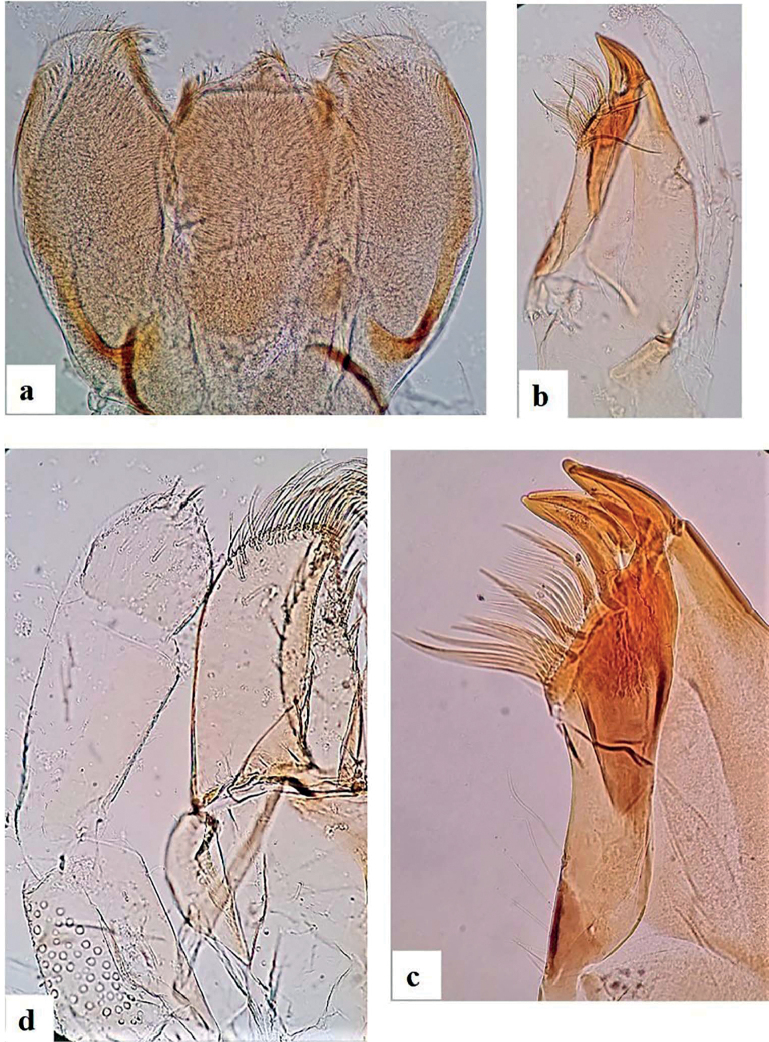
*Alainitesalbai* sp. nov., nymph morphology **a** hypopharynx **b** maxilla **c** details of dentisetae and maxilla crown of setae **d** labium.

***Hypopharynx*** (Fig. 5a) trilobed, apically covered with thin setae; lingua with small central protuberance; superlingua longer than lingua.

***Maxilla*** (Fig. 5b) apex with three elongated acute and curved teeth and a toothlike dentisetae; crown with one row of small setae ending with stouter and longer ones (Fig. 5c); palp two-segmented, extending behind apex of galea-lacinia, length of segment I approximately 0.65× segment II; segment II apically rounded, with few thin setae.

***Labium*** (Fig. 5d) with glossae slightly shorter than paraglossae; inner margins of glossae with 7 or 9 stout medium setae, apical margin with ca 5–7 long stout setae, ventral surface with few thin scattered setae; dorsal surface with row of ca 6–9 medium setae; paraglossae of constant width, with three rows of 11 or 14 long, stout and feathery setae apically; labial palp three-segmented; segment I 0.8× length of segments II and III combined; segment II with dorsal oblique row of 4–6 medium setae; segment III conical, nearly symmetrical, slightly pointed apically, covered with few medium stout setae and small pointed setae apically.

**Figure 6.**
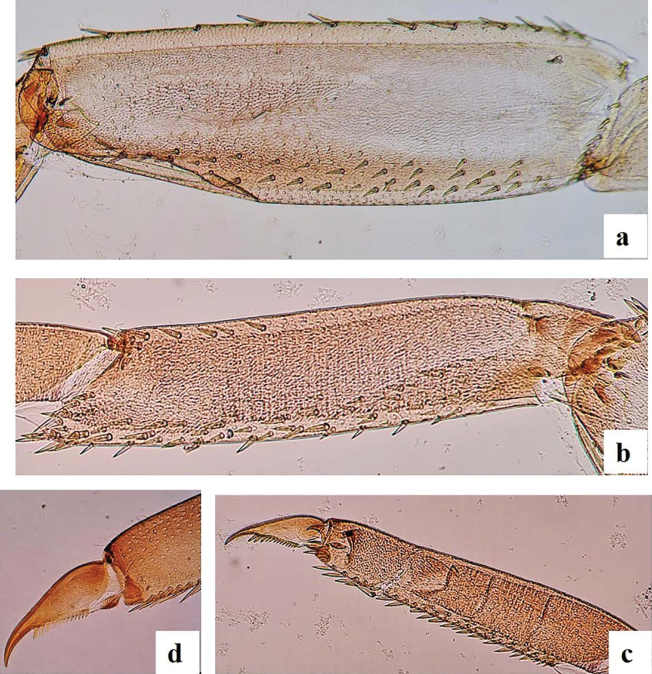
*Alainitesalbai* sp. nov., nymph morphology **a** foreleg femur **b** foreleg tibia **c** foreleg tarsal claw, foreleg claw.

***Thorax*. *Forelegs*** (Fig. 6): Trochanter with seven marginal short stout pointed setae; femur dorsal margin with one regular row of 10–15 long, robust setae, and two subapical stout and long setae; ventral margin with short stout pointed setae; lateral surface with sparse scale bases, mainly on apical half and along subdorsal area (Fig. 6a). Tibiae (Fig. 6b) dorsally with 6–10 short stout pointed setae and single apical seta; ventrally with marginal and submarginal short stout pointed setae, denser towards apical end; tibiopatellar suture absent; lateral surface with few short, stout, pointed setae and numerous scale bases. Tarsi bares dorsally; ventral margin with ca 15–24 pointed medium setae; lateral surface with numerous scale bases. Tarsal claws (Fig. 6c) hooked with one row of 11–13 medium teeth, apical setae absent (Fig. 6d). Mid and hindlegs similar to forelegs except femora dorsally with 13–17 pointed setae and tibiae with 21–24 setae dorsally.

***Hindwing.*** Pads present.

**Figure 7.**
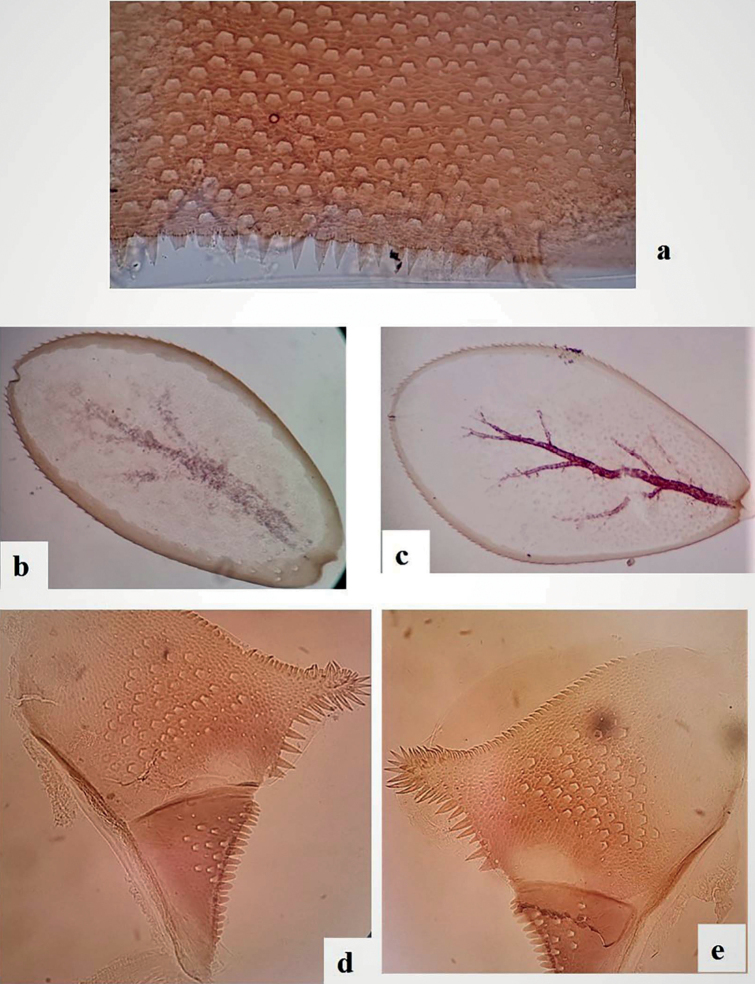
*Alainitesalbai* sp. nov., nymph morphology **a** distal margin of tergum IV **b** gill IV **c** gill V **d** left paraproct **e** right paraproct.

***Abdomen*. *Terga*** (Fig. 7a) shagreened, with numerous scale bases, distal margin of tergite IV with triangular spines ~ 2× longer than broad. Sternites with scale bases; posterior margin smooth without spination.

***Gills*** (Fig. 2d) on segments I–VII, elliptic, almost symmetrical and serrated all along costal and distal margins except proximal part and with few setae, gill I smaller than others, length of gill I longer than half of tergite II; gill IV (Fig. 7b) and gill V larger than others, (Fig. 7c). Surface with pores and without any setae; tracheation well visible but with reduced ramification. Paraproct (Fig. 7d, e) shagreened with scale bases and setae; margin with seven broad, triangular spines inner to prolongation and numerous medium spines outer to prolongation; lateral margins of prolongation with numerous medium to broad spines, surface covered with spines; cercotractor with scattered scale bases, distal margin with ca 20 medium spines.

**Imago.** Unknown.

#### Molecular analysis.

**Figure 8.**
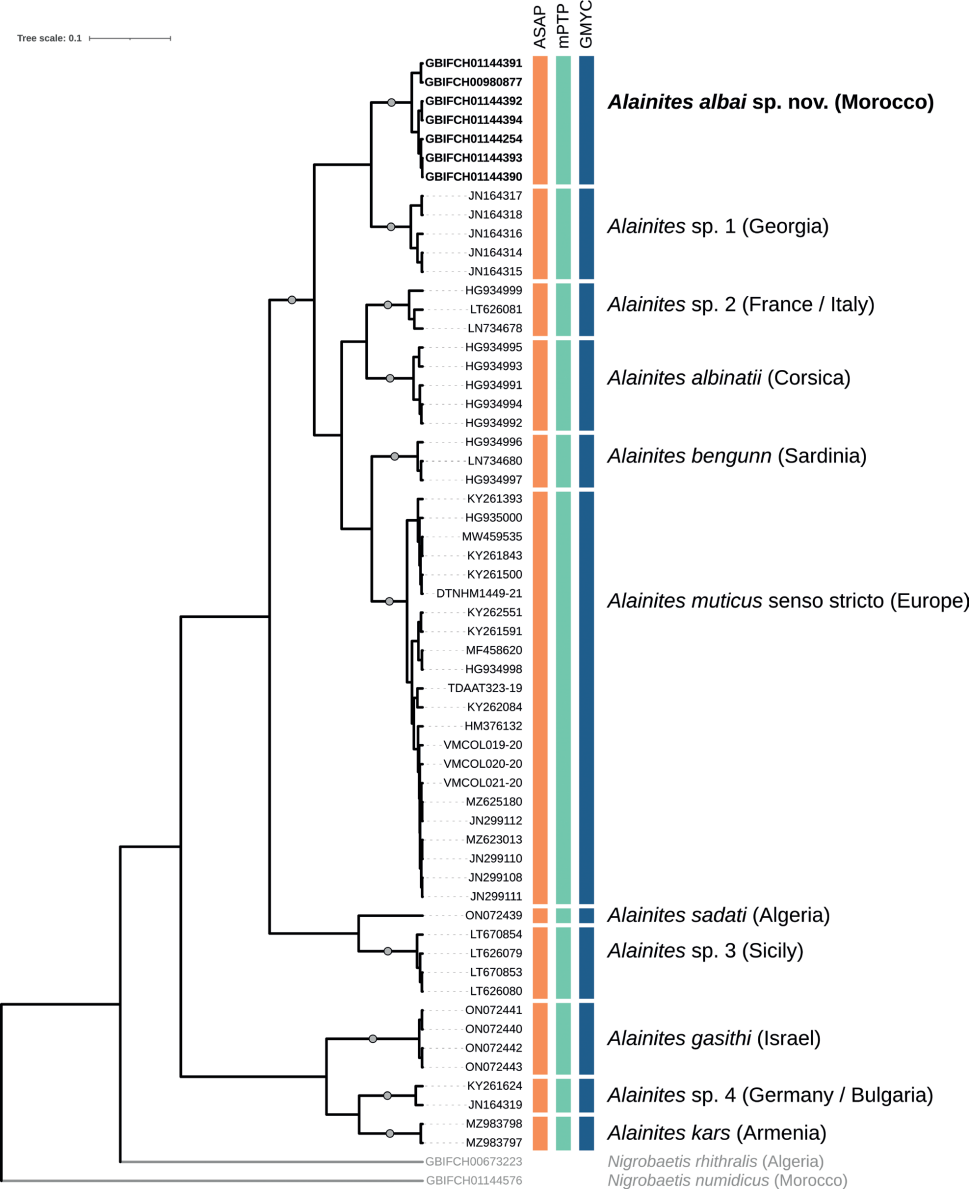
Bayesian (BEAST) maximum clade credibility COI tree of the genus Alainites in the West Palearctic: branch ends labelled with GBIF codes in bold indicate newly sequenced specimens; the DTNHM1449-21, TDAAT323-19, and VMCOL019-20–VMCOL021-20 codes are from BOLD; the other codes correspond to sequences obtained from GenBank. Colored vertical boxes indicate species delimitation hypothesis according to the ASAP, mPTP, and GMYC methods. For each MOTU, the corresponding species names (where available) and the country/region of origin is provided, with the newly described species and associated GBIF codes specified in bold. Circles on branches indicate Bayesian posterior probabilities > 0.9. Outgroup branches, labels, and species names are presented in grey.

The COI data set was > 95% complete and included 35% of parsimony informative sites. Pairwise COI distances across all sequences ranged from 0% to 23.4%. The overall mean p-distance within MOTUs was 0.8% (mean range: 0.1%–2.3%), while the overall mean p-distance between MOTUs was 18.7% (mean range: 14.9%–22.6%). The maximum p-distance within MOTUs varied from 0.2% (*A.gasithi* and *A.kars*) to 2.8% (*A.* sp. 2). The minimum p-distance between MOTUs ranged from 14.3% (*A.* sp. 1–*A.* sp. 2) to 16.7% (*A.kars*–*A.gasithi* and *A.kars*–*A.* sp. 4). The seven sequences from *A.albai* sp. nov. formed a strongly supported monophyletic clade, identified as a distinct MOTU in the three species delimitation analyses (Fig. 8). All methods were fully congruent in delimitating the other MOTUs as well.

#### Distribution

(Fig. 1). Morocco.

#### Etymology.

The first author dedicates the name of this species to her former mentor, Professor Javier Alba-Tercedor, a specialist in the Ephemeroptera of the Iberian Peninsula. He contributed significantly to her training and specialization in the taxonomic study of the Ephemeroptera of Morocco.

## ﻿Discussion

### ﻿Morphology and genetic characteristics

The integrative approach, incorporating molecular, morphological, and biogeographic evidence, enabled a robust species delimitation within *Alainites*. Indeed, the molecular analyses not only support the monophyly of *Alainitesalbai* sp. nov. (Fig. 8), but also reveal an important genetic distance to all other included *Alainites* species. The minimum p-distance to the other MOTUs ranges from 16% to 20%, thereby solidifying *A.albai* sp. nov. as a distinct and valid new species. The findings from our molecular analyses also underline the need for further taxonomic research on *Alainites*, particularly in Europe.

The number of gills is a useful character to separate the different species of *Alainites*. This new species is assigned to *Alainites* because it has all the synapomorphic characters of the genus: a laterally compressed thorax, paraproct with postero-internal extension and the prostheca of the right mandible bifid (Waltz et al. 1994; Gattolliat et al. 2023). Within *Alainites*, the larva of the new species is the second western Palearctic species with seven pairs of gills. It differs from *A.muticus* by the size of the spines between prostheca and mola of the right mandible, by the reticulation degree of paraprocts, tergites and mandibles, by the spination of the paraproct extension surface and the presence of strong spines between the prolongation and the extension of the paraproct. *Alainitesalbai* sp. nov. can be easily separated from the other western Palearctic species (*A.albinatii*, *A.bengunn*, *A.gasithi*, *A.navasi*, *A.kars*), as well as the Maghreb endemic species (*A.oukaimeden* and *A.sadati*) by the number of pairs of gills (Table 2).

Hence, the discovery of this new species has increased the diversity of described *Alainites* species with seven pairs of gills to a total of six, with two found in the West Palaearctic and four in the Oriental realms. The latter are *A.siamensis* Phlai-ngam, Tungpairojwong & Gattolliat, 2022 from Thailand (Phlai-ngam et al. 2022), *A.lingulatus* Tong & Dudgeon, 2000 from Hong Kong (Tong and Dudgeon 2000), *A.yixiani* Gui & Lu, 1999 from the Chinese mainland (Gui and Lu 1999; Sroka et al. 2021; Phlai-ngam et al. 2022), and *A.clivosus* Chang & Yang, 1994 from Taiwan (Kang et al. 1994; Waltz et al. 1994; Kluge and Novikova 2014; Sroka et al. 2021).

### ﻿Ecology

*Alainitesalbai* sp. nov. was first mentioned in the Rif and Middle Atlas by Dakki and El Agbani (1983) under the name *Baetismuticus*. This new species is confined preferentially to streams of northern Morocco, since it appears to be absent from the High Atlas (Bouzidi 1989; El Alami et al. 2022b), where it is replaced by its congener *A.oukaimeden*. Its absence in eastern Morocco (Berrahou et al. 2001; Mabrouki et al. 2019) and the Central Plateau (El Agbani et al. 1992) is probably related to excessive water heating.

In addition to the wide horizontal distribution of this species in northern Morocco, it has a fairly wide altitudinal distribution (5–1600 m) covering the three bioclimatic stages, the thermos-, meso-, and supra-Mediterranean. In fact, it shows a preference for the streams along the Mediterranean coastline with a semi-arid climate over regions with a sub-humid to humid climate. In certain mountainous regions it can thrive in conditions considered as perhumid. In the Rif, this species likes biotopes with a stony bottom rich in sand and submerged vegetation. In addition, it prefers the relatively cold waters of the upper and middle courses of wadis in which it reaches its ecological optimum during the summer period. In the Middle Atlas, its distribution is more restricted, as it has only been collected in two wadis at altitudes varying between 760 and 1500 m (Dakki 1987). However, recent surveys have shown the species to now be absent from these stations (Zerrouk et al. 2021).

## ﻿Conclusions

The discovery of *A.albai* sp. nov. has increased the biodiversity of Morocco with a new endemic species of mayfly, highlighting the remarkable biodiversity and species richness of Ephemeroptera in the region. The recent identification of two new species, *Prosopistomamaroccanum* (El Alami et al., 2022a) and *Centroptilumalamiae* (Gattolliat et al., 2023), has significantly increased the proportion of Ephemeroptera endemism in Morocco to more than 33% (El Alami et al. 2022b). With the potential for further increases in the future, it is crucial to prioritize conservation and protection measures, particularly in sites hosting high species richness and endemic species.

## Supplementary Material

XML Treatment for Alainites albai

## ﻿Additional information

### Author ORCIDs

Majida El Alami https://orcid.org/0000-0003-2664-646X

Laurent Vuataz https://orcid.org/0000-0001-9193-8683

Sara El Yaagoubi https://orcid.org/0000-0003-1860-6433

Jean-Luc Gattolliat https://orcid.org/0000-0001-5873-5083

### Data availability

All of the data that support the findings of this study are available in the main text.
